# Comparative Genomic Analysis of Meningitis- and Bacteremia-Causing Pneumococci Identifies a Common Core Genome

**DOI:** 10.1128/IAI.00814-15

**Published:** 2015-09-10

**Authors:** Benard W. Kulohoma, Jennifer E. Cornick, Chrispin Chaguza, Feyruz Yalcin, Simon R. Harris, Katherine J. Gray, Anmol M. Kiran, Elizabeth Molyneux, Neil French, Julian Parkhill, Brian E. Faragher, Dean B. Everett, Stephen D. Bentley, Robert S. Heyderman

**Affiliations:** a Malawi-Liverpool-Wellcome Trust Clinical Research Programme, University of Malawi College of Medicine, Blantyre, Malawi; b Institute of Infection and Global Health, University of Liverpool, Liverpool, United Kingdom; c The Wellcome Trust Sanger Institute, Wellcome Trust Genome Campus, Hinxton, Cambridge, United Kingdom; d University of Malawi College of Medicine, Blantyre, Malawi; e Liverpool School of Tropical Medicine, Liverpool, United Kingdom

## Abstract

Streptococcus pneumoniae is a nasopharyngeal commensal that occasionally invades normally sterile sites to cause bloodstream infection and meningitis. Although the pneumococcal population structure and evolutionary genetics are well defined, it is not clear whether pneumococci that cause meningitis are genetically distinct from those that do not. Here, we used whole-genome sequencing of 140 isolates of S. pneumoniae recovered from bloodstream infection (*n* = 70) and meningitis (*n* = 70) to compare their genetic contents. By fitting a double-exponential decaying-function model, we show that these isolates share a core of 1,427 genes (95% confidence interval [CI], 1,425 to 1,435 genes) and that there is no difference in the core genome or accessory gene content from these disease manifestations. Gene presence/absence alone therefore does not explain the virulence behavior of pneumococci that reach the meninges. Our analysis, however, supports the requirement of a range of previously described virulence factors and vaccine candidates for both meningitis- and bacteremia-causing pneumococci. This high-resolution view suggests that, despite considerable competency for genetic exchange, all pneumococci are under considerable pressure to retain key components advantageous for colonization and transmission and that these components are essential for access to and survival in sterile sites.

## INTRODUCTION

*Streptococcus pneumoniae* is estimated to cause almost 15 million episodes of pneumonia, meningitis, and bloodstream infection in children under five annually, with the highest burden in sub-Saharan Africa (SSA) (1). An important HIV-related pathogen, the pneumococcus is also associated with a significant number of deaths in HIV-infected African adults (2). Nasopharyngeal colonization is a prerequisite for pneumococcal disease, which follows a complex multistep process of bloodstream and less frequently cerebrospinal fluid (CSF) invasion through a combination of engagement between bacteria and host epithelial and endothelial receptors, cytoskeletal rearrangement and transcytosis, and infection of migrating phagocytes (3–6). The avoidance of immune surveillance and the capacity of S. pneumoniae to grow and survive in the relatively hostile blood and meningeal compartments facilitate these processes (3, 7). Why some patients develop pneumococcal bacteremia but not meningitis while in others infections occur in both compartments is uncertain.

Our fundamental understanding of the bacterial mechanisms that determine these host-pathogen interactions is at best incomplete and is supported largely by data from tissue culture models and animal studies (4, 5, 8). In particular, specific pneumococcal determinants that explain the tropism of this capsulated bacterium for the brain have been elusive. Whether pneumococci that cause meningitis are genetically distinct from those that do not is uncertain. While some pneumococcal capsule serotypes are particularly associated with invasive pneumococcal diseases (IPD) in humans (9, 10), each with a different invasive potential, there is currently no evidence that either the serotypes or sequence types (STs) that cause bacteremia and meningitis are distinct (11). Likewise, numerous other bacterial molecules have been implicated in the pathogenesis of pneumococcal meningitis (e.g., phosphorylcholine, pneumolysin, pneumococcal surface adhesin A, neuraminidase A, and pneumococcal adherence and virulence factor A), but whether these are absolutely required for meningeal invasion and disease causation remains to be determined. Adaptation of the pneumococcus to the host environment is facilitated by mutations and by frequent transfers of genetic material (horizontal gene transfer) between isolates and across bacterial species (12–14). This genetic variability provides considerable redundancy in the range of tools available to S. pneumoniae to target host receptors, overcome the mucosal barrier, and survive within the nasopharynx (7, 15–17).

Here, we have used whole-genome sequencing of a large collection of disease-causing isolates to revisit pneumococcal meningitis in humans. We have specifically identified genes that are universally present in pneumococci associated with invasive disease (the pneumococcal core genome) and those that are present only in some invasive isolates (the pneumococcal accessory genome) and have investigated the hypothesis that meningitis-causing isolates have a genetic content distinct from isolates associated with bloodstream infection, which explains their ability to survive in these hostile host environments.

## MATERIALS AND METHODS

### Strain selection, DNA extraction, and genome sequencing.

S. pneumoniae isolates selected for genome sequencing (see Table S1 in the supplemental material) were obtained from the Malawi-Liverpool-Wellcome Trust Clinical Research Programme (MLW) strain archive. Isolates collected between 2002 and 2008 (*n* = 140) were randomly selected from the archive isolate database using STATA v 9.2 (StataCorp, College Station, TX). MLW has conducted externally quality-controlled surveillance for bacterial meningitis and bloodstream infection at Queen Elizabeth Central Hospital, Blantyre, Malawi, for over 15 years (1–3). The inclusion criteria, culture rate, and laboratory methods have not changed appreciably over this period. Pneumococcal isolates were obtained from the CSF of children and adults where meningitis was suspected (*n* = 70) and blood of children and adults with suspected bloodstream infection but not meningitis (*n* = 70) (1, 3). Children were defined as patients <15 years of age. HIV status was not available for the isolates. Too few paired isolates for a robust analysis were available and therefore were not used. Likewise, too few noninvasive isolates from Malawi were available to allow a robust comparison of noninvasive to invasive (blood/CSF) isolates; therefore noninvasive isolates were not included in this analysis.

Isolates were grown overnight in Todd-Hewitt broth (Oxoid limited, Basingstoke, Hampshire, England), and genomic DNA was isolated from the bacteria using a Wizard genomic DNA purification kit (Promega, USA; product number A1125). Multiplex library construction, with a 200-bp insertion size and 54-nucleotide (nt) or 108-nt paired-end Illumina sequencing, followed by a third 7-nt tag read, was performed according to standard protocols (13, 18). Serotype and sequence type data were derived from genome sequences using previously described protocols (13). The isolates used in the study were anonymized. These data are published with the approval of the University of Malawi College of Medicine Research & Ethics Committee and conform to institutional guidelines.

### Assembly of draft genomes and annotation.

*De novo* assembly of draft genome sequences was achieved from multiplexed Illumina sequence data as previously described (13, 18). Briefly, assemblies were made using Velvet v 0.7.03 (19). Velvet was optimized to run with the longest *k*-mer that gave an expected coverage value above 20 (13). The draft genome assemblies were ordered against the S. pneumoniae ATCC 700669 genome (see Table S2 in the supplemental material) using ABACAS (20) and ACT (21). Coding sequences (CDS) were identified using Glimmer3 (22).

### Genome clustering and core genome analysis.

Gene annotations from 12 publicly available, complete, fully annotated reference genomes (see Table S2 in the supplemental material) were transferred onto the CDS in the draft genomes using RATT (23). Annotated genes were translated to protein sequences and assigned to orthologous gene “clusters” using tribeMCL (24) with default parameters (BLASTP E value cutoff of 1e−5 and inflation index of 4) (full details of the clustering pipeline can be found at https://github.com/fy2/pneumoscript). The OrthoMCL output was used to establish the core genome for the data sets.

The tribeMCL ortholog clusters were organized into a matrix of genome content using bespoke Perl scripts, with orthologs from the same genome arranged in columns and rows identifying annotated orthologs with similar functions. A blank was inserted where an ortholog was absent. Genomes (the matrix columns) were randomly sampled in an arithmetic progression fashion according to the equation *S_N_ = N/*2[2*a +* (*N* − 1)*d*]. The number of random sampling events, *S_N_*, was established using the least number of genomes under consideration, *a* (i.e., 1 genome in this study), the total number of genomes under consideration (i.e., the data set size), *N*, and the common difference of successive genomes, *d* (i.e., 1) to be used during sampling. During random sampling, *N* was initialized as 1 genome for the first event and increased by one unit for each of the subsequent events, until it was equivalent to the total number of genomes in the data set in the final event. The random genomes were sampled only once during each event, and the total orthologs shared by all genomes were counted once for each cluster, thereby excluding paralogous counts. This was iterated 100 times, resulting in 100 input orders for each event; the arithmetic average core genome size was computed for each event to enable the average core genome size to be related to the number of genomes sampled. Comparisons of core genomes from different data sets were achieved using Perl scripts and STATA v 9.2 (Stata Corp, College Station, TX).

### Core genome extrapolation.

The absolute core genome size was extrapolated by fitting exponentially decaying functions as previously described by Tettelin et al. (29). For our data, two distinct decay phases were identified, so the following double-exponential model was fitted to the data using the Levenberg-Marquardt iterative estimation method: *y* = kc_1_ · exp(−*n*/tc_1_) + kc_2_ · exp(−*n*/tc_2_) + gamma. In this model, *y* is the size of the core genome, *n* is the number of sequenced strains, kc_1_ and tc_1_ are the exponent and free parameters, respectively, for the first decay phase, kc_2_ and tc_2_ are the exponent and free parameters, respectively, for the second decay phase, and gamma is the predicted value of the core genome size with an infinite number of sequenced strains.

## RESULTS

### The pneumococcal core genome.

We first set out to define the pneumococcal core genome, i.e., those genes that are common to all human invasive pneumococcal disease isolates. Hiller and colleagues have previously undertaken a similar analysis using a data set of 17 pneumococcal genomes (eight from nasopharyngeal isolates from pediatric patients and nine invasive and avirulent isolates that were publically available). They found that 1,454 gene clusters (46%) were conserved among all isolates (25). Here, we have subjected to Illumina sequencing 140 randomly selected invasive pneumococcal isolates, which originate from Malawian adults and children with meningitis (*n* = 70) or bloodstream infection without clinical meningitis (bacteremia; *n* = 70). The HIV status of the individuals from which the isolates were recovered was not available. These isolates were collected as part of routine invasive bacterial surveillance at a large teaching hospital in Malawi (2, 26, 27). The 140 genomes had a mean sequencing read depth of 155× (minimum depth of 38×) (full details of assembly statistics are listed in Table S4 in the supplemental material). The isolates belong to different serotypes and clonal types as determined by multilocus sequence typing (MLST) (see Table S1 in the supplemental material) and consequently vary in their previously reported association with invasive disease (28). The genomes were first *de novo* assembled using Velvet v 0.7.03 (19); prediction of coding sequences and functional annotation were done using Glimmer3 and RATT (22, 23). A mapping approach, which involves the alignment of query genome sequence reads against a single reference genome, would not have been be suitable for this analysis, as mapping highly recombinogenic clones from multiple diverse lineages against a single reference genome would considerably restrict the degree of variation detected.

**FIG 1:**
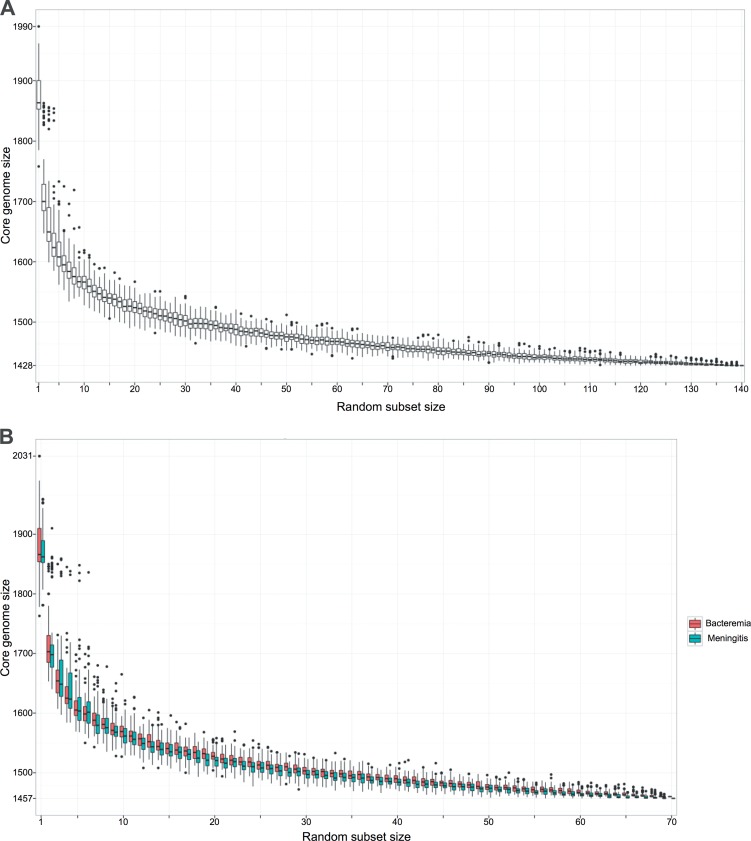

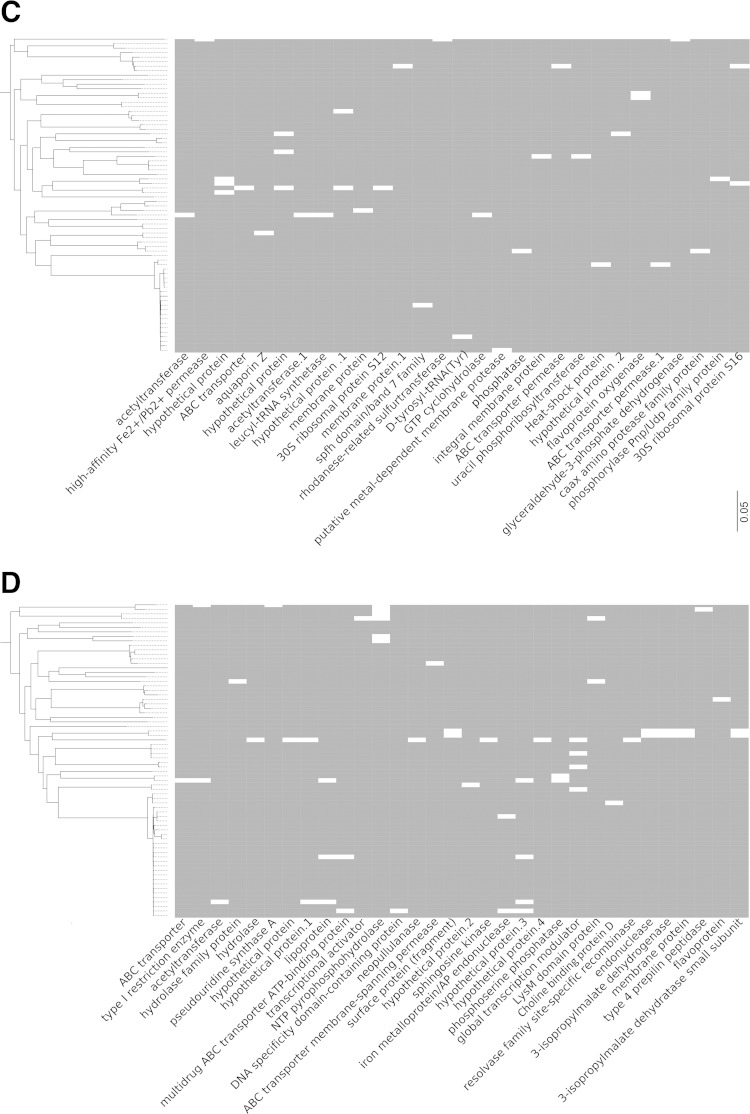
Meningitis- and bacteremia-associated pneumococcal core genomes based on 140 invasive isolates. (A) Box and whisker plot of the number of core gene orthologous clusters observed as the subset of isolates included in the analysis increases, where the subset ranged from 1 to the total number of isolates (*n* = 140). To generate a subset of isolates, *n* isolates are randomly selected from the data set. The core genome size is then calculated for *n* isolates. Each random subset of a given size is generated 100 times. (B) Box and whisker plot of the number of meningitis and bacteremia core gene orthologous clusters observed as the number of isolates included in the analysis increases, where the subset ranged from 1 to the total number of isolates (*n* = 70). To generate a subset of isolates, *n* isolates are randomly selected from the data set. The core genome size is then calculated for *n* isolates. Each random subset of a given size is generated 100 times. (C) Map of the proportion of meningitis-specific core genes in the bacteremia-associated pneumococcal data set (genes present and absent are represented in gray and white, respectively). A list of meningitis-specific proteins is on the *x* axis, and individual bacteremia-specific isolates with a phylogenetic tree are on the *y* axis. (D) Map of the proportion of bacteremia-specific genes in the meningitis-associated pneumococcal data set (genes present and absent are represented in gray and white, respectively). A list of bacteremia-specific proteins is on the *x* axis, and individual meningitis isolates with a phylogenetic tree are on the *y* axis.

We identified orthologous genes in the assembled genome by the process of clustering based on homology. The clustering of orthologous genes provided a robust basis for comparing and combining data from multiple diverse genomes. Following clustering, genes from all isolates were divided into three categories: orthologous genes shared by all isolates (core genes), orthologous genes shared between two or more isolates (accessory genes), and genes unique to one isolate. We identified 3,164 orthologous gene clusters in all 140 isolates. Forty-five percent of the total clusters (1,428 core genes) were present in all of the study isolates (Fig. 1; see Table S3 in the supplemental material). Of the remaining clusters, 1,612 (51%) were present in two or more isolates, but not all isolates, while 124 genes (4%) did not group within a cluster and were unique to a single isolate. Of the core genes, 1,239/1,428 (87%) could be assigned a definitive function; the remaining 189 core genes (13%) were either of unknown or putative function.

A considerable amount of care was taken to ensure that the genome assemblies were of good quality and therefore did not distort the outputs by applying a stringent quality control (QC) process. Nonetheless, poor assembly of small genomic regions may have resulted in legitimate core genes being wrongly defined as accessory genes in our analysis, thus causing us to marginally underestimate the core genome size. Relaxation of the stringency to allow for this possibility, whereby a core gene was defined as an orthologous gene shared by ≥98% or more isolates, marginally increased the core genome (the “extended core genome”) size to 1,469 genes. As reported previously, the core genome size was dependent on the number of isolates used during the analysis and gradually decreased with the addition of new genomes (Fig. 1A) but did not reach an absolute plateau, as has been reported in studies of group B Streptococcus that examined smaller data sets (29). To ascertain if we had reached a definitive core genome size for S. pneumoniae, we undertook a decaying-function analysis as previously described (29). However, instead of a single-exponential decaying function, we found that we could extrapolate the data until it plateaued with a higher degree of precision by fitting a double-exponential decaying function (see Fig. S1 in the supplemental material). Consistent with the core genome size observed in our analysis of 140 genomes, we found that, when extrapolated to infinity using the double-exponential decaying function, the core genome reached a minimum of 1,427 genes (95% confidence interval [CI] 1,425 to 1,435 genes; *R*^2^ = 99.6). It can therefore be concluded that the data set employed in this study allowed us to accurately assess the core genome content of pneumococci isolated in Malawi.

### Composition of the core genome.

The core genome common to all 140 human invasive pneumococcal disease isolates contains many genes that have been implicated in nasopharyngeal colonization by inhibiting mucus function, reducing niche competition by other bacterial species, subverting host cell function, and facilitating host cell attachment (Table 1; for a complete list of core genes see Table S3 in the supplemental material). In animal models, many of these virulence genes have also been shown to facilitate tissue invasion and promote disease. The virulence factors encoded by the overall core genome identified here include: β-galactosidase (BgaA) and β-*N*-acetylglucosaminidase (StrH), which deglycosylate mucus glycoconjugate to reduce mucus viscosity, thereby limiting mucus entrapment and exposing host cell surface adherence receptors that are exploited by the pneumococcus for attachment (3, 30), and pyruvate oxidase (SpxB), which catalyzes the production of hydrogen peroxide, which kills niche competitors to facilitate pneumococcal colonization (8, 15). In view of the report that serotype 1 patient isolates from the ST217 clonal complex can have spontaneous mutations in *spxB* that seem in increase virulence (4), we investigated allelic variation in the *spxB* gene in the data set. We found the SpxB protein to be highly conserved, and no allelic variants of *spxB* were unique to either the bacteremia or the meningitis isolates.

**TABLE 1:** Summary of key proteins associated with the pneumococcal core genome

Category	Core gene products
Antimicrobial resistance	ABC transporter antimicrobial extrusion family protein, MATE
	Multidrug resistance protein, MdtG
Transporters	Manganese efflux system, SP1552
	ABC transporter, BlpC
	Cobalt ABC transporter, CbiO
Virulence factors	β-Galactosidase, BgaA
	BlpC (quorum-sensing pheromone), which controls Blp
	Choline binding protein A, CbpA
	Choline binding protein D, CbpD
	Choline binding protein E, CbpE
	Choline binding protein G, CbpG
	Choline binding protein H, CbpH
Competence regulation	Competence protein A, ComA
	Competence protein D, ComD
	Competence protein E, ComE
	Competence protein X, ComX
	Autolysin, LytA
	Cell wall hydrolase, LytB
	Cell wall hydrolase, LytC
	Neuraminidase, NanB
	β-*N*-Acetylglucosaminidase, StrH
	Serine/threonine protein kinase
DNA synthesis, repair, and regulation	Superoxide dismutase, SodA
	DNA repair protein, RecN
	DNA mismatch protein, RadC
	DNA inducible damage protein D

Also identified in the overall core genome were genes encoding virulence proteins that enhance colonization either through binding host ligands or subverting immune defense, including pneumolysin (Ply) (3, 8, 17), autolysin (LytA) (17), choline-binding protein E (CbpE) (5, 16), pneumococcal adhesion and virulence A (PavA) (3, 16), and immunoglobulin A1 protease (IgA1 protease). The overall core genome contains genes that enable niche adaptation and regulation of metabolism in response to alterations in available carbon sources, cations and amino acids, and anaerobic conditions (31). These include the formate acetyltransferase gene (*pfl*), which is essential for fermentative metabolism. Pfl mutants have reduced virulence, evidenced by prolonged survival times in mouse models of bacteremia (31). Fe^2+^ ATP-binding cassette (ABC) transporters, which are members of the ABC transporter superfamily, translocating a variety of substrates such as cations, lipids, and amino acids across membranes (32), have been specifically implicated in pneumococcal virulence (33). Previous PCR-based studies have shown that several of the associated genes, while present in all of our invasive isolates, are not always present in carriage isolates (34, 35). For example, genes encoding NanB and CbpA have been found to be present in only 81% and 54% of carriage pneumococci screened, respectively (35). This implies that carriage isolates encode an alternative set of proteins that aid colonization, suggesting that there is considerable redundancy in the range of tools available to the pneumococcus for survival in the nasopharyngeal niche.

Once the pneumococcus has reached the bloodstream, invasion of the meninges is thought to require a series of genes that mediate host cell attachment and transcytosis through the blood-brain barrier (BBB) and then survival within the CSF (3, 4, 8). Since bacteremia and meningitis are a dead end for the pneumococcus, it is unlikely that these core genes have evolved solely to facilitate invasion of the brain. We speculate that these core genes are important for niche survival at the mucosal surface but also facilitate invasion into and survival in the blood and CSF. Ply disrupts BBB integrity, PavA facilitates endothelial fibronectin engagement, and Hyl degrades the endothelial extracellular matrix; in animal models all three of these functions have been strongly implicated in the pathogenesis of meningitis. We identified a number of other genes that have been specifically implicated in pneumococcal meningitis in the overall core genome. These include genes encoding choline binding protein A (CbpA, SpsA, or PspC), a member of a family of surface proteins implicated in colonization (17), which binds to the polymeric immunoglobulin receptor (pIgR) in order to cross from the mucosal surface into the blood (3, 36). In the absence of pIgR on the surface of brain endothelium, CbpA binds laminin to initiate contact and translocation across the BBB (3, 4). The core genome also encodes pneumococcal competence proteins (ComA, ComD, ComE, and ComX), which regulate biofilm formation and are associated with virulence in animal models of meningitis (30, 37). The prominence of DNA repair and regulation genes in the overall core genome identified by our analysis suggests the critical nature of gene variation for pneumococcal survival in a variety of environments (12, 38).

Some of the genes identified in the common core genome also encode putative pneumococcal protein antigen vaccine candidates currently under evaluation (Table 1), with the notable exception of pilin and NanA, whose genes were not present in the core genome (3, 8, 17, 39). Other key virulence factors whose genes were not present in the core genome include pneumococcal histidine triad protein B (PhtB), pneumococcal histidine triad protein E (PhtE), pneumococcal surface protein A (PspA), neuraminidase A (NanA), neuraminidase C (NanC), pilus subunit A (RrgA), pilus subunit B (RrgB), pilus subunit C (RrgC), and bacteriocin (Blp). Mahdi and colleagues recently conducted a genome-wide *in vivo* transcriptomic analysis of pneumococci reaching the brain after murine intranasal infection (40). They identified a previously uncharacterized protein, alpha-glycerophosphate oxidase (GlpO), which was substantially upregulated in brain tissue. GlpO was cytotoxic for human brain microvascular endothelial cells (HBMECs), and immunization of mice with GlpO protected against invasive pneumococcal disease. The gene encoding GlpO was present in the overall core genome (Table 1). The finding of these putative vaccine candidates in the overall core genome is especially attractive as it suggests that a vaccine based on these candidate molecules is likely to be protective against a wide range of invasive pneumococcal strains.

### The core genomes of bloodstream infection and meningitis pneumococcal isolates.

We then conducted separate analyses of the core genomes of the pneumococcal bloodstream and meningitis subsets to test the hypothesis that there are distinct gene sets that are absolutely required for invasion, growth, and persistence in these different niches. The core genomes of the bloodstream and meningitis subsets were very similar in size (1,457 versus 1,460 genes, respectively) (Fig. 1B). Our analysis identified a subset of 32 genes that were present in the bloodstream core genome but absent in the meningitis core genome (meningitis-specific core genes) and a subset of 29 genes that were present in the meningitis core genome but absent in the bloodstream core genome (bloodstream-specific core genes). However, the genetic differences identified by this comparative analysis (Fig. 1C and D) were due to the absence of key genes in just 3/70 (4%) of the bloodstream isolates (Fig. 1C) and 4/70 (6%) of the meningitis isolates (Fig. 1D). We found no impact of imbalanced sampling leading to the dominance of a particular clone or an overrepresentation of prophage and integrative conjugative elements (ICE) on the results of the comparative analysis. Therefore, given the known biological functions of the genes identified and the relatively small proportion of isolates responsible for the differences, we think it unlikely that the core genome of meningitis-causing pneumococci is truly distinct from the core genome of bloodstream isolates.

### The accessory genomes of bloodstream infection and meningitis pneumococcal isolates.

**FIG 2:**
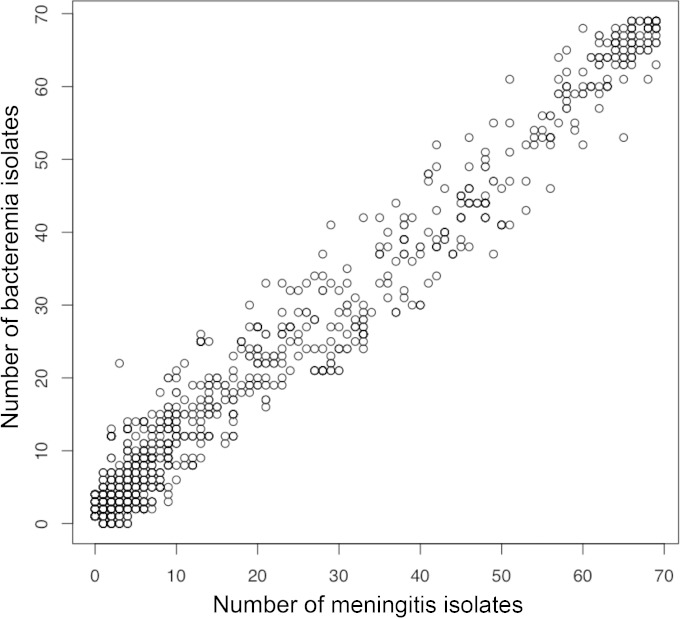
Plot of the number of meningitis and bacteremia isolates represented in each accessory gene cluster. Each circle represents a single gene cluster.

We next compared the accessory genomes of the bloodstream and meningitis isolates to interrogate the possibility that a large proportion of the bloodstream isolates possess a subset of accessory genes that were not present in the meningitis accessory genome (Fig. 2). No accessory gene clusters were composed of only bloodstream or meningitis isolates. Among the accessory gene clusters there was a strong positive correlation between the number of bloodstream isolates and the number of meningitis isolates represented in a cluster (Spearman's correlation = 0.79; *P* < 0.0001). No accessory genes were more strongly associated with the bacteremia or meningitis data set.

### Factors influencing the pneumococcal core genome.

The pneumococcal isolates analyzed were randomly selected to avoid a selection bias, and given the size of the data set, there should be a clonal balance representative of the invasive strains in circulation. However, in many SSA countries, serotype 1 (ST217) accounts for over 30% of IPD (9, 10), and in our randomly selected Malawian meningitis data set it accounted for 24% (33/140) of isolates (14 ST217 isolates recovered from blood, 19 isolates recovered from CSF). It has been suggested that highly virulent serotype 1 pneumococcal isolates have genomic regions associated with pathogenicity that are absent in others that are intermediately virulent or avirulent (41). Therefore, to address the possibility that “genetic noise” from the other sequence types (STs) present in the data set may have masked legitimate differences in the core genome composition between ST217 causing bacteremia and ST217 causing CSF invasion, we repeated the above analyses using the ST217 clones alone. The core genomes of the ST217 bloodstream and meningitis subsets differed in size (1,822 versus 1,795 genes, respectively), and we identified a subset of 42 genes that were present in the ST217 bloodstream core genome but absent in the ST217 meningitis core genome (ST217 meningitis-specific core genes) and a subset of 15 genes that were present in the ST217 meningitis core genome but absent in the ST217 bloodstream core genome (ST217 bloodstream-specific core genes). However, as with the observed core genome differences between the bacteremia and meningitis causing isolates in the overall core genome analysis, the difference in the ST217 core genome sizes was due to the absence of key genes in only a very small number of isolates. The 42 core genes that were unique to the ST217 bacteremia isolates were at most absent in 4/19 (≤21%) of the ST217 meningitis isolates; likewise the 15 core genes that were unique to the ST217 bacteremia isolates were absent in at most 2/14 (≤14%) of the ST217 bacteremia isolates. Again, as with the whole data set, we next compared the accessory genomes of the ST217 meningitis and bloodstream isolates to assess if a large proportion of the ST217 meningitis isolates possess a subset of accessory genes that were not present in the ST217 bloodstream accessory genome. We found that no ST217 accessory gene clusters consisted only of bloodstream or meningitis isolates. We observed a positive correlation between the number of ST217 meningitis isolates and the number of ST217 bloodstream isolates represented in an accessory gene cluster (Spearman's rank coefficient = 0.84; *P* < 0.0001); this strongly suggests that no ST217 accessory genes were more strongly associated with a specific invasive disease phenotype.

We next carried out the same analysis with all ST217 isolates excluded and found that there was no difference between the genome compositions of the bacteremia and meningitis isolates when ST217 was removed from the data set. The core genomes of the bacteremia and meningitis isolates, with ST217 excluded, comprised 1,467 and 1,463 genes, respectively. The 29 bacteremia “unique” isolates were absent in at most 3/53 (≤6%) of the meningitis isolates; the meningitis “unique” isolates were absent in at most of 4/45 (≤8%) of the bacteremia isolates. We observed a positive correlation between the number of meningitis isolates and the number of bloodstream isolates represented in an accessory gene cluster when the ST217 isolates were excluded from the analysis (Spearman's rank coefficient = 0.92; *P* < 0.0001). This additional analysis based on subsets of the data set strongly suggests that our initial finding, which reported that there is no difference in the core genome content between bacteremia and meningitis S. *pneumoniae* isolates, is not an artifact of sample bias.

## DISCUSSION

Numerous human host factors that determine pneumococcal disease susceptibility and severity have been identified, including extremes of age, HIV, splenic dysfunction, sickle cell disease, malignancy, chronic liver disease, malnutrition, and diabetes (42, 43). In the past our understanding of the bacterial components contributing to disease was largely supported by tissue culture models and animal studies. Here, we have used an innovative bioinformatics approach and a stringent analysis pipeline to interrogate the pneumococcal genome associated with invasive disease. We have found, by fitting a double-exponential decaying function, that disease isolates share a core of 1,427 genes (95% CI, 1,425 to 1,435 genes). The core genome size we report is marginally lower than the core genome size reported for Streptococcus pneumoniae by Obert et al. (*n* = 1,553) (44) and Hiller et al. (*n* = 1,454) (25). However, the reduced core genome size can be explained by the higher number of isolates and serotypes included in our analyses, which have likely resulted in greater saturation and a more precise estimate.

We also show that pneumococci that infect the meninges do not differ in their core or accessory genome content from those isolated from the blood, and therefore gene presence/absence alone does not explain the virulence behavior of some pneumococci that reach the brain. Although the development of meningitis may simply be a function of bacterial load and time (26, 45–48), the potential effect of more-subtle differences at the genomic level, specifically allelic variation and gene expression, requires further investigation. For example, a comparison of the genomic sequences of four nasopharyngeal PMEN1 isolates and their capacities to cause disease in a chinchilla otitis media model revealed allelic differences in Blp regulatory proteins and pneumococcal adherence virulence factor B (PavB) in those isolates that spread to the brain (49). Gerlini et al. have shown that bacteremia is a result of a single pneumococcal clone invading the blood; this clone then disseminates, and the resulting invasive pneumococcal population acquires genomic mutations over time as it adapts to the selective pressures of the bloodstream. It is feasible that the same behavior is true for meningitis, whereby a single clone crosses the BBB, proliferates, and rapidly acquires genomic mutations to permit survival in the CSF (50). Microarray analysis of pneumococcal gene expression from blood and CSF infected *in vitro* has previously identified site-specific patterns of gene expression (51). Furthermore, host differences identified by numerous genetic association studies have shown that single nucleotide polymorphisms (SNPs) in innate immunity genes are linked to an individual's susceptibility to pneumococcal meningitis. This suggests that specific pneumococcal genotypes may be able to cause meningitis in some individuals but not others, irrespective of the core genome composition and SNP profile of that genotype (52–54).

A limitation of the study was that it was not possible from the clinical data available to determine if any of the bacteremia isolates included in the data set went on to invade the meninges. It is therefore feasible that some meningitis isolates were incorrectly assigned as bacteremia isolates. This may have masked subtle differences in the accessory genome composition between the bacteremia and meningitis isolates, whereby accessory genes that are more strongly associated with meningitis isolates were found to be evenly distributed between the meningitis and bacteremia isolates in our analysis. This, however, will not have affected the overall core genome composition reported here. It is feasible that the ability of pneumococci to cause meningitis is dictated by the presence of a subset of meningitis-specific genes linked through function and that there may be more than one subset combination that confers the ability to cause meningitis. This hypothesis was not addressed in the current analysis.

The finding of a repertoire of over 1,400 core genes that are common to all invasive-disease-causing pneumococci despite the highly recombinogenic nature of the multiple diverse lineages investigated provides an important biological insight. The presence of these core genes and their associated proteins in all disease-causing isolates suggests that they are presumably advantageous for colonization and transmission, as well as being essential for the pathogen to invade and survive within the bloodstream and meninges. They include a range of previously identified vaccine candidate molecules and a number of the core gene clusters (13%) that could not be assigned a definitive function. The further characterization of these genes together with an assessment of allelic variation and gene expression among pneumococci from different sites may also reveal important new bacterial housekeeping functions and identify novel candidates that can be exploited as vaccine and therapeutic targets.

## Supplementary Material

Supplemental material
