# Supplementary material for: Comparative Genomic Analysis of Meningitis- and Bacteremia-Causing Pneumococci Identifies a Common Core Genome
Source: Infect Immun. 2015 Sep 10;83(10):4165–73. doi: 10.1128/IAI.00814-15 (PMC4567637; doi:10.1128/IAI.00814-15)
Supplement: Supplemental material [file supp_83_10_4165__index.html]

Comparative Genomic Analysis of Meningitis- and Bacteremia-Causing Pneumococci Identifies a Common Core Genome — Supplemental material 

# Comparative Genomic Analysis of Meningitis- and Bacteremia-Causing Pneumococci Identifies a Common Core Genome

## Supplemental material

- Supplemental file 1 -

  Fig. S1. Extrapolation of the core genome sizes using the double-exponential model. Table S1. Sequenced isolates used in this study. Table S2. Complete, fully annotated genomes used as references. Captions to Tables S3 and S4.

  PDF, 233K
- Supplemental file 2 -

  Table S3. Entire list of the common core genes to the meningitis and bacteremia dataset.

  XLSX, 95K
- Supplemental file 3 -

  Table S4. Genome assembly statistics for the sequenced isolates used in this study.

  XLSX, 82K
