# Supplementary material for: Comparative Genomic Analysis of Meningitis- and Bacteremia-Causing Pneumococci Identifies a Common Core Genome
Source: Infect Immun. 2015 Sep 10;83(10):4165–73. doi: 10.1128/IAI.00814-15 (PMC4567637; doi:10.1128/IAI.00814-15)
Supplement: Supplemental material [file IAI.00814-15_zii999091430so1.pdf]

Supporting Information legends

Figure S1. Extrapolation of the core genome sizes using the double-exponential model. The data was extrapolated by fitting a double exponential decaying function to evaluate the absolute number of genes in study isolates. The overall core genome reached a minimum of 1427 genes (95% CI 1420 to 1435; $R^2 = 99.6$) when extrapolated to infinity.

Table S1. Sequenced isolates used in this study. The sample accession and run accession are given in the first and second columns. The next five columns give the diagnostic lab sample ID followed by details of the patient age, the serotype of the isolate, and the source from which the isolate was obtained (CSF for meningitis and blood for bacteremia). The last column shows the MLST.

Table S2. The complete, fully annotated genomes used as references.

Table S3. Entire list of the common core genes to the meningitis and bacteremia dataset. Due to redundancy in naming orthologs several gene names and gene identities are provided. All orthologs that belong to the same cluster have the same gene cluster number

Table S4. Genome assembly statistics for the sequenced isolates used in this study.

Figure S1.

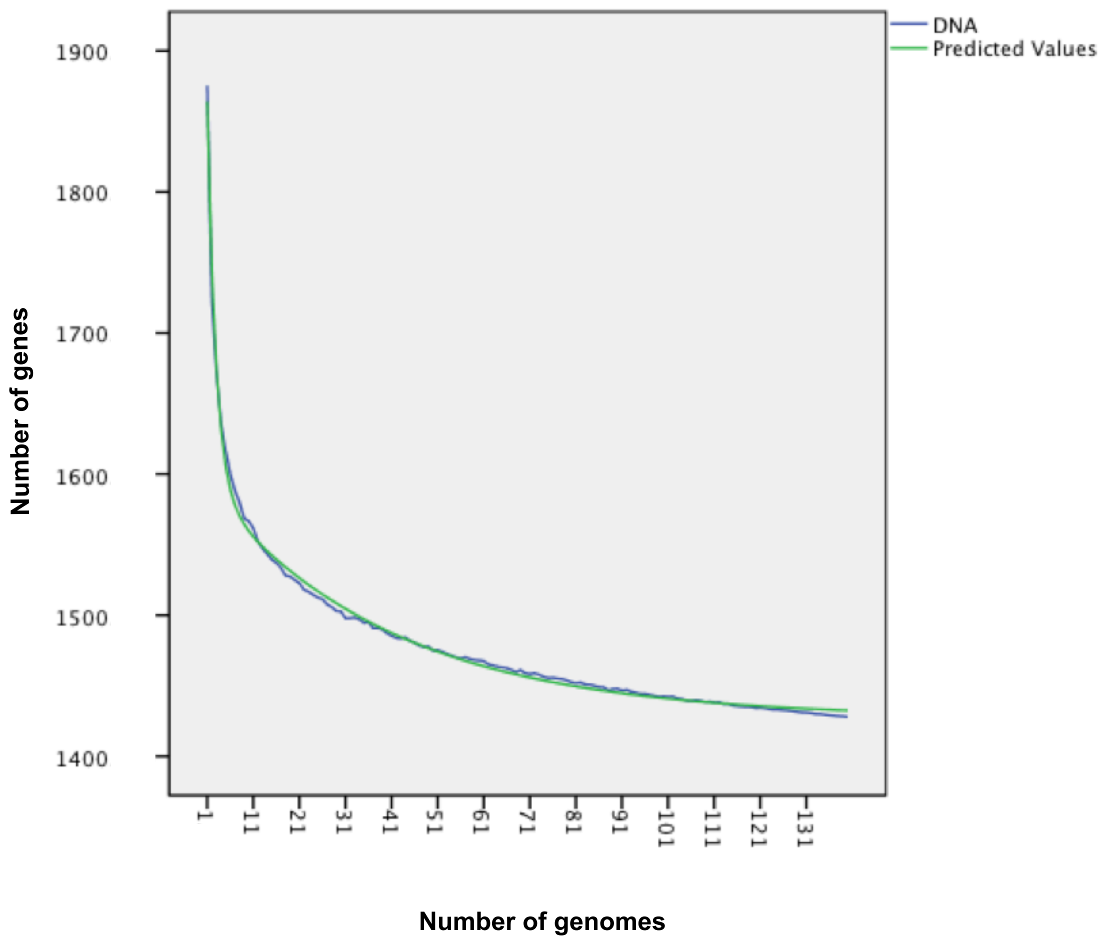

Estimate				
kc_1	tc_1	kc_2	tc_2	gamma
470.927	1.841	395.183	40.023	1427.464

Table S1.

Sample Accession	Run Accession	Sample ID	Individual	Serotype	Source	MLST
**ERS005601**	ERR023349	A32245	Adult	1	Blood	217
**ERS005602**	ERR023321	D31094	Child	1	Blood	217
**ERS005613**	ERR023331	D28368	Child	1	Blood	217
**ERS005626**	ERR045783	D28324	Child	1	Blood	217
**ERS005627**	ERR023345	D36913	Child	1	Blood	217
**ERS005633**	ERR045787	D38050	Child	1	Blood	217
**ERS012093**	ERR028422	A28816	Adult	1	Blood	217
**ERS012120**	ERR028438	A33973	Adult	1	Blood	217
**ERS012121**	ERR028439	A34030	Adult	1	Blood	217
**ERS024510**	ERR033122	D34485	Child	1	Blood	217
**ERS050335**	ERR068176	A31105	Adult	1	Blood	217
**ERS050342**	ERR068183	A31584	Adult	1	Blood	217
**ERS050346**	ERR068187	A32572	Adult	1	Blood	217
**ERS050496**	ERR086047	D35438	Child	1	Blood	217
**ERS003569**	ERR023977	B17099	Adult	1	CSF	217
**ERS005597**	ERR023358	C14599	Child	1	CSF	217
**ERS005612**	ERR023330	C15160	Child	1	CSF	217
**ERS005614**	ERR023322	C14269	Child	1	CSF	217
**ERS005620**	ERR023340	C13632	Child	1	CSF	217
**ERS005638**	ERR023337	B16942	Adult	1	CSF	217
**ERS006734**	ERR018021	C14099	Child	1	CSF	217
**ERS006743**	ERR018042	C16000	Child	1	CSF	217
**ERS006745**	ERR018044	C13215	Child	1	CSF	217
**ERS006746**	ERR018034	C13181	Child	1	CSF	217
**ERS006820**	ERR023389	C14249	Child	1	CSF	217
**ERS012073**	ERR028411	B14935	Adult	1	CSF	217
**ERS012088**	ERR028427	B17333	Adult	1	CSF	217
**ERS026047**	ERR033141	C12907	Child	1	CSF	217
**ERS050414**	ERR085965	B17224	Adult	1	CSF	217

**ERS050415**	ERR085966	B17789	Adult	1	CSF	217
**ERS050416**	ERR085967	B17998	Adult	1	CSF	217
**ERS050417**	ERR085968	B18042	Adult	1	CSF	217
**ERS050453**	ERR086004	C12797	Child	1	CSF	217
**ERS003550**	ERR023964	A33056	Adult	1	Blood	303
**ERS006824**	ERR023393	C11020	Child	1	CSF	303
**ERS005629**	ERR045786	D28572	Child	1	Blood	8158
**ERS005590**	ERR023352	A31470	Adult	1	Blood	Novel
**ERS005618**	ERR023338	D33216	Child	1	Blood	Novel
**ERS012122**	ERR028440	A34045	Adult	1	Blood	Novel
**ERS050334**	ERR068175	A30992	Adult	1	Blood	Novel
**ERS050494**	ERR086045	D27102	Child	1	Blood	Novel
**ERS003575**	ERR023983	B15567	Adult	1	CSF	Novel
**ERS005594**	ERR023348	C11680	Child	1	CSF	Novel
**ERS005598**	ERR023357	B13691	Adult	1	CSF	Novel
**ERS005603**	ERR023326	C12458	Child	1	CSF	Novel
**ERS005611**	ERR023329	B10261	Adult	1	CSF	Novel
**ERS005615**	ERR023323	B10454	Adult	1	CSF	Novel
**ERS005624**	ERR023343	C14737	Child	1	CSF	Novel
**ERS008357**	ERR024531	C15514	Child	1	CSF	Novel
**ERS006727**	ERR018025	D31621	Child	10B	Blood	705
**ERS012094**	ERR026611	A29037	Adult	10B	Blood	7055
**ERS008345**	ERR024523	B14457	Adult	10B	CSF	7055
**ERS012087**	ERR028426	B16827	Adult	10B	CSF	7055
**ERS026044**	ERR033144	C11367	Child	10B	CSF	7055
**ERS050418**	ERR085969	B18107	Adult	10B	CSF	7055
**ERS012097**	ERR026616	A29943	Adult	12B	Blood	989
**ERS012099**	ERR026618	A31131	Adult	12B	Blood	989
**ERS003564**	Novel	B15071	Adult	12B	CSF	989
**ERS012076**	ERR028414	B15249	Adult	12B	CSF	989
**ERS026056**	ERR033118	C15284	Child	12B	CSF	989
**ERS003548**	ERR023966	B13048	Adult	12B	CSF	Novel
**ERS006804**	ERR018108	B16221	Adult	12B	CSF	Novel
**ERS050457**	ERR086008	C14971	Child	12B	CSF	Novel
**ERS050338**	ERR068179	A31198	Adult	12F	Blood	989

**ERS006802**	ERR018106	B17731	Adult	12F	CSF	989
**ERS012086**	ERR028425	B16392	Adult	13	CSF	2053
**ERS050456**	ERR086007	C14277	Child	13	CSF	2053
**ERS005609**	ERR023327	D28370	Child	13	Blood	5647
**ERS026043**	ERR033145	D32344	Child	13	Blood	Novel
**ERS006736**	ERR018037	D25696	Child	14	Blood	63
**ERS012092**	ERR028421	A28640	Adult	14	Blood	63
**ERS050495**	ERR086046	D27950	Child	15A	Blood	Novel
**ERS012102**	ERR026621	B10622	Adult	15C	CSF	Novel
**ERS012142**	ERR028461	D30716	Child	16F	Blood	705
**ERS026050**	ERR033138	D37909	Child	16F	Blood	705
**ERS006818**	ERR023387	D28531	Child	18A	Blood	Novel
**ERS012113**	ERR026633	D28166	Child	18B	Blood	102
**ERS024520**	ERR033126	C10281	Child	18B	CSF	102
**ERS026048**	ERR033140	C13260	Child	18B	CSF	Novel
**ERS008361**	ERR024535	D33733	Child	18F	Blood	Novel
**ERS050452**	ERR086003	C12675	Child	18F	CSF	Novel
**ERS050341**	ERR068182	A31361	Adult	19A	Blood	2062
**ERS050340**	ERR068181	A31349	Adult	19A	Blood	Novel
**ERS026040**	ERR033137	D32289	Child	19F	Blood	Novel
**ERS006738**	ERR018036	D26316	Child	23F	Blood	802
**ERS012116**	ERR028432	A33308	Adult	23F	Blood	802
**ERS012141**	ERR028460	D30625	Child	23F	Blood	802
**ERS012075**	ERR028413	B15188	Adult	23F	CSF	802
**ERS026058**	ERR033116	C15291	Child	23F	CSF	802
**ERS050420**	ERR085971	B18340	Adult	23F	CSF	802
**ERS008320**	ERR024496	A32273	Adult	23F	Blood	Novel
**ERS050343**	ERR068184	A31918	Adult	23F	Blood	Novel
**ERS008335**	ERR024512	B10366	Adult	23F	CSF	Novel
**ERS050345**	ERR068186	A32497	Adult	24F	Blood	5077
**ERS050454**	ERR086005	C12879	Child	24F	CSF	5077
**ERS050333**	ERR068174	A29265	Adult	24F	Blood	Novel
**ERS006733**	ERR018031	D38094	Child	25F	Blood	2902
**ERS012112**	ERR026632	D24847	Child	25F	Blood	2987
**ERS006732**	ERR018030	C14376	Child	25F/A	CSF	Novel

**ERS026041**	ERR033136	D36881	Child	3	Blood	700
**ERS050433**	ERR085984	B20605	Adult	3	CSF	700
**ERS008359**	ERR024533	D31827	Child	34	Blood	Novel
**ERS050339**	ERR068180	A31265	Adult	35B	Blood	5396
**ERS012071**	ERR028398	B14721	Adult	35B	CSF	5396
**ERS012119**	ERR028437	A33922	Adult	4	Blood	2213
**ERS050344**	ERR068185	A32279	Adult	4	Blood	2213
**ERS006737**	ERR018035	C14560	Child	4	CSF	5483
**ERS003574**	ERR023982	B15467	Adult	45	CSF	Novel
**ERS050419**	ERR085970	B18210	Adult	45	CSF	Novel
**ERS012095**	ERR026614	A29101	Adult	5	Blood	289
**ERS050641**	ERR085862	D48490	Child	5	Blood	289
**ERS003576**	ERR023984	B16728	Adult	5	CSF	289
**ERS006744**	ERR018043	C15085	Child	5	CSF	289
**ERS050646**	ERR085867	D55377	Child	5	Blood	Novel
**ERS008360**	ERR024534	D33293	Child	6A	Blood	2987
**ERS008351**	ERR024529	C11950	Child	6A	CSF	2987
**ERS008352**	ERR024519	C12495	Child	6A	CSF	2987
**ERS012078**	ERR028416	C14215	Child	6A	CSF	2987
**ERS008347**	ERR024525	B16242	Adult	6A	CSF	5412
**ERS012145**	ERR028464	D36051	Child	6A	Blood	Novel
**ERS026049**	ERR033139	D36355	Child	6B	Blood	2285
**ERS003549**	ERR023967	A33096	Adult	6B	Blood	Novel
**ERS012143**	ERR028462	D33275	Child	6B	Blood	Novel
**ERS050336**	ERR068177	A31122	Adult	6B	Blood	Novel
**ERS050451**	ERR086002	C12480	Child	6B	CSF	Novel
**ERS012083**	ERR028420	B15901	Adult	6C	CSF	2902
**ERS050441**	ERR085992	B21980	Adult	6D	CSF	2902
**ERS012079**	ERR028417	D38023	Child	6D	Blood	Novel
**ERS050428**	ERR085979	B19805	Adult	7F	CSF	Novel
**ERS050347**	ERR068188	A32849	Adult	7f	Blood	Novel
**ERS012124**	ERR028442	A34562	Adult	9A	Blood	1871
**ERS024518**	ERR033128	C10300	Child	9A	CSF	1871
**ERS012123**	ERR028441	A34292	Adult	9A	Blood	5778
**ERS012096**	ERR026615	A29167	Adult	9A	Blood	5902

**ERS012098**	ERR026617	A30277	Adult	9A	Blood	5902
**ERS012100**	ERR026619	B10027	Adult	9A	CSF	5902
**ERS012117**	ERR028435	A33813	Adult	9A	Blood	Novel
**ERS024513**	ERR033133	D30974	Child	9A	Blood	Novel
**ERS050409**	ERR085960	B10199	Adult	9L	CSF	Novel
**ERS050455**	ERR086006	C14257	Child	9L	CSF	Novel

Table S2.

Genome no.	GenBank accession number	Other name	Serotype	Sequencing centre	Year	Reference
**1**	AE005672	TIGR4	4	TIGR	2001	Tettelin H et al. Science 293 (5529):498-506 (2001)
**2**	AE007317	R6	NT	Eli Lilly and Company	2001	Hoskins J et al. J Bacteriol 183(19), 5709-5717, 2001
**3**	CP000410	D39	2	TIGR	2004	Lanie J.A et al. J Bacteriol 189(1), 38-51, 2007
**4**	CP000936	Hungary 19A-6	19A	J. Craig Venter Institute	2008	Hotopp,J.D. et al. Direct submission. J. Craig Venter Institute
**5**	CP001015	G54	19F	Institute for Genome Sciences	2008	Dopazo J et al. Microb Drug Resist 7(2), 99-125, 2001
**6**	CP001033	CGSP14	14	Beijing institute of Genomics	2008	Ding F et al. BMC Genomics 10, 158, 2009
**7**	CP000918	70585	5	J. Craig Venter Institute	2009	Hotopp,J.D. et al. Direct submission. J. Craig Venter Institute
**8**	CP000919	JJA	14	J. Craig Venter Institute	2009	Hotopp,J.D. et al. Direct submission. J. Craig Venter Institute
**9**	CP000920	P1031	1	J. Craig Venter Institute	2009	Hotopp,J.D. et al. Direct submission. J. Craig Venter Institute
**10**	CP000921	Taiwan 19F-14	19F	J. Craig Venter Institute	2009	Hotopp,J.D. et al. Direct submission. J. Craig Venter Institute
**11**	FM211187	ATCC700 669	23F	Wellcome Trust Sanger Institute	2009	Croucher N.J. et al. J Bacteriol 191(5), 1480-1489, 2009
**12**	CP001993	670	6B	Baylor College of Medicine	2010	Muzny,D.M. et al. Direct submission. Baylor College of Medicine
